# The complete chloroplast genome sequence of *Lepidium perfoliatum* L.

**DOI:** 10.1080/23802359.2021.1872437

**Published:** 2021-03-01

**Authors:** Qian Zhou, Wei-Jun Zeng, Ji-Lian Wang, Yu-Zhou Wang, Yun Chen, Kaidiriye Yusupul, Hui-Xin Zhao

**Affiliations:** a College of Life and Geography Sciences, Key Laboratory of Biological Resources and Ecology of Pamirs Plateau in Xinjiang Uygur Autonomous Region, Kashi University, Kashi, China; b Xinjiang Key Laboratory of Special Species Conservation and Regulatory Biology, Key Laboratory of Plant Stress Biology in Arid Land, College of Life Science, Xinjiang Normal University, Urumqi, China

**Keywords:** *Lepidium perfoliatum* L., chloroplast genome, phylogenemic tree

## Abstract

This study was based on the collection of the complete genome of *Lepidium perfoliatum* chloroplast (cp). The full cp genome is 154,264 bp long, containing 130 genes, in which 8 genes are specified for ribosomal RNA (rRNA), while 85 and 37 genes for protein-coding and transfer RNA (tRNA) respectively. Phylogenetic analyss revealed the closed cluster of *Lepidium perfoliatum* with other *Lepidium* species such as *Lepidium apetalum*, *Lepidium sativum*, *Lepidium meyenii* and *Lepidium virginicum*, which helps for the evaluation of how *Lepidium perfoliatum* is phylogenetically related to other species.

*Lepidium perfoliatum* L. is a short-lived annual desert plant in the cruciferous family, naturally distributed in Xinjiang, Liaoning, Jiangsu, Gansu, and other places in China (Meng et al. 2008). Whole grass can be used as a medicine, with diuresis and anti-scurvy effect, etc. In addition, *L. perfoliatum* is also a kind of high-quality forage, with a unique physiological mechanism to adapt to the desert and early spring environment (Yang et al. 2015). In recent years, studies on the genus *L. perfoliatum* have focused on the characteristics of seed slime (Huang et al. 2015), total protein extraction (Ding et al. 2017), endophytic bacteria (Li et al. 2017), and salt tolerance (Roghieh et al. 2018). The genus *Lepidium* has been a relatively specialized group. Due to the lack of sufficient identification characteristics, the classification of genus *Lepidium* has always been difficult (Sun and Li 2007). However, no prior reports of the *L. perfoliatum* chloroplast genome are available, it may be important to elucidate the evolution of cruciferae species.

Fresh *L. perfoliatum* leaves were obtained from Carp mountain (Xinjiang, China, 87°34′716″E, 43°50′577″N) in Urumqi, Xinjiang, China and deposited the Voucher specimens in the Xinjiang Normal University herbarium (No. SHFZDXC02). Total extraction of gDNA was conducted via modified CTAB approach (Li et al. 2013) which was then utilized for building the Illumina pair-end library and its sequencing was carried out with an Illumina HiSeq platform (Illumina, CA, USA) at Tianjin Genomics Institute (TGS-Shenzhen, China) and yielded the raw data of approximately 4.4 GB. High-quality data contig assembly was then conducted with the SPAdes v3.9.0 de novo assembler (Bankevich et al. 2012), followed by futher assembly into an overall cp genome with NOVOPlast2.7.1 (Dierckxsens et al. 2017). Following assembly, these data were compared to the published *L. sativum* (MN176145) complete cp genome of. All cp gene annotation was conducted with the DOGMA tool using default parameters (Wyman et al. 2004).We have deposited the annotated cp genome of *L. perfoliatum* in Genbank under accession number MT880913.1.

The overall size of the *L. perfoliatum* cp genome is 154,264 bp with 36.46% GC content, which consists of 83,391 bp large single-copy region (LSC), 17,973 bp small single-copy region (SSC), and 26,450 bp inverted repeat regions pairs (IRA and IRB). It was predicted that the chloroplast genome consists of 130 unique genes, which includes 85 protein-coding genes, 8 rRNA, and 37 tRNA genes.

Of all the unique genes, 15 genes (*trnK-UUU*, *trnL*-*UAA*, *rps16*, *trnG*-*UCC*, *atpF*, *trnV*-*UAC*, *rpoC1*, *ndhB*, *petB*, *rpl16*, *petD*, *rpl2*, *trnA*-*UGC*, *trnI*-*GAU*, *ndhA*) contained a single intron, while 2 genes (*clpP*, *ycf3*) contained two introns. Similarly, one trans-splicing gene (*rps12* gene) was also found. Phylogenic maximum-likelihood (ML) trees, based on a complete cp genome of 21 species which were obtained from the GenBank database, were constructed by using MAGE 7.0 (Kumar et al. 2016). Phylogenetic analysis indicated *L. perfoliatum* was sister to *L. apetalum*, *L. sativum*, *L. meyenii,* and *L. virginicum*, within *Lepidium* (Figure 1), which is analogous to previously reported data (Zhu et al. 2019). Our study here could be further applied for the evolutionary and phylogenetic studies of this Brassicaceae Burnett plant.

**Figure 1.**
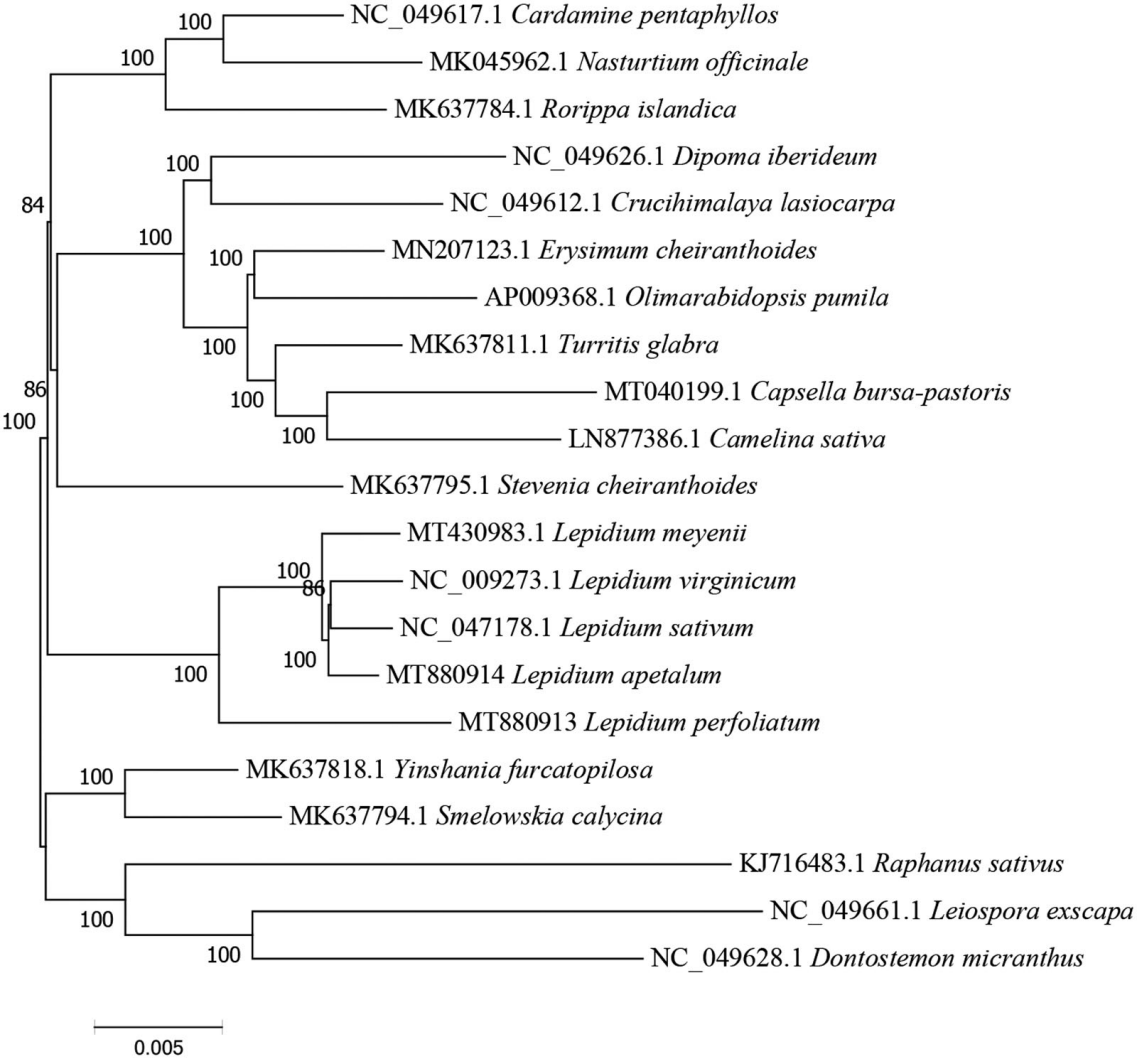
Complete cp genome data (21 species) based ML phylogenetic tree was constructed.

## Data Availability

The data that support the findings of this study are openly available in GenBank of NCBI at https://www.ncbi.nlm.nih.gov, reference number MT880913.1. The associated BioProject, SRA, and Bio-Sample numbers are PRJNA682689, SRR13213156, and SAMN16993569 respectively.
